# The complete mitochondrial genome of the golden apple snail *Pomacea canaliculata* (Gastropoda: Ampullariidae)

**DOI:** 10.1080/23802359.2015.1137816

**Published:** 2016-03-23

**Authors:** Huirong Yang, Jia-en Zhang, Zhixin Deng, Hao Luo, Jing Guo, Simei He, Mingzhu Luo, Benliang Zhao

**Affiliations:** a College of Animal Science, South China Agricultural University, Guangzhou, China;; b Institute of Tropical and Subtropical Ecology, South China Agricultural University, Guangzhou, China;; c Key Laboratory of Agro-Environment in the Tropics, Ministry of Agriculture P. R. China, Guangzhou, China;; d Guangdong Provincial Engineering Technology Research Center of Modern Eco-agriculture and Circular Agriculture, Guangzhou, China

**Keywords:** Ampullariidae, mitochondrial genome, *Pomacea canaliculata*

## Abstract

We present the complete mitochondrial genome of *Cipangopaludina cathayensis* in this study. The mitochondrial genome is 15 706 bp in length, containing 13 protein-coding genes, two rRNA genes and 22 tRNA genes. Overall nucleotide compositions of the light strand are 40.97% of A, 30.78% of T, 20.48% of C and 12.60% of G. Its gene arrangement and distribution are different from the typical vertebrates. The absence of D-loop is consistent with the Gastropoda, but, at least, one lengthy non-coding region is an essential regulatory element for the initiation of transcription and replication. A phylogenetic tree is constructed using the maximum-likelihood method based on the complete mitogenomes of the closely related 21 Gastropoda species to assess their actual phylogenetic relationship and evolution. The result provides fundamental data for resolving phylogenetic and genetic problems related to effective management strategies.

The golden apple snail *Pomacea canaliculata* (Lamarck 1822) indigenous to South America, was introduced from Argentina to Taiwan for commercial purposes, subsequently to numerous countries throughout southern and eastern Asia including China, becoming pests of wetland rice and other crops and causing massive economic losses. They also continue to spread into non-agricultural wetlands and their ecological impacts are more difficult to estimate (Wood et al. 2005; Levin et al. 2006; Rawlings et al. 2007). The United States Aquatic Nuisance Species Task Force listed *P. canaliculata* among the world’s 100 worst invasive species (Lowe et al. 2000). But, the confused taxonomy and difficult identification of genus Pomacea results from the overall highly conserved external morphology across the genus yet considerable intraspecific shell variation, obscuring the true number of species and their identities (Thiengo et al. 1993; Cazzaniga 2002; Cowie 2002; Cowie et al. 2006).

We sequenced its complete mitogenome to analyze phylogenetic relationship and evolutionary history for broader understanding of invasion processes and implementing effective management strategies. The specimen was sampled from Ningxi Teaching Experimental Base of South China Agricultural University in Guangzhou (E 113°29′, N 23°5′), and stored in the specimen museum of SCAU (accession number: 201502116).

The complete mitochondrial genome of *P. canaliculata* (GenBank accession number KU052865) is 15 706 bp in length, containing 13 protein-coding genes, two ribosomal RNA genes (L-rRNA and S-rRNA), 22 transfer RNA genes (tRNA). The rest of them are encoded on the heavy strand except eight tRNA genes (Met, Tyr, Cys, Trp, Gln, Gly, Glu, Thr) on the light strand. Twenty-two tRNA genes vary from 62 to 70 bp in length, and all fold into the typical cloverleaf secondary structure. Among 13 protein-coding genes (total 11 220 bp) encoding 3727 amino acids, the maximum is ND5 with 1710 bp and the minimum is ATP8 with only 159 bp. S-rRNA and L-rRNA genes are 877 and 1381 bp, respectively, located between the tRNA^Glu^ and tRNA^Leu^ genes and separated by the tRNA^Val^ gene. Overall nucleotide compositions of the light strand in descending order are 40.97% of A, 30.78% of T, 20.48% of C and 12.60% of G. Gene arrangement and distribution are different from the typical vertebrates (Yang et al. 2014a, 2016a–g) and similar to *Cipangopaludina cathayensis* (Yang et al. 2014b). The absence of D-loop is consistent with the Gastropoda (Liu et al. 2012; Zeng et al. 2015; Zhou et al. 2016), but, at least, one lengthy non-coding region is an essential regulatory element for the initiation of transcription and replication (Wolstenholme 1992).

A phylogenetic tree is constructed using the maximum-likelihood method based on the complete mitogenomes of the closely related 21 Gastropoda species to assess their actual phylogenetic relationship and evolution (Figure 1). But repeat elements (AAAGAAACTAAGAGATAAGATAT)N and (AGTTTCTTTATATCTTATCTCTT)N which are located between tRNA-Phe (GAA) and COX3 gene pair into the complex hairpin structure to prevent the PCR process. So, it is difficult to verify the accurate information by Hiseq2500 sequencing system and needs further research.

**Figure 1.**
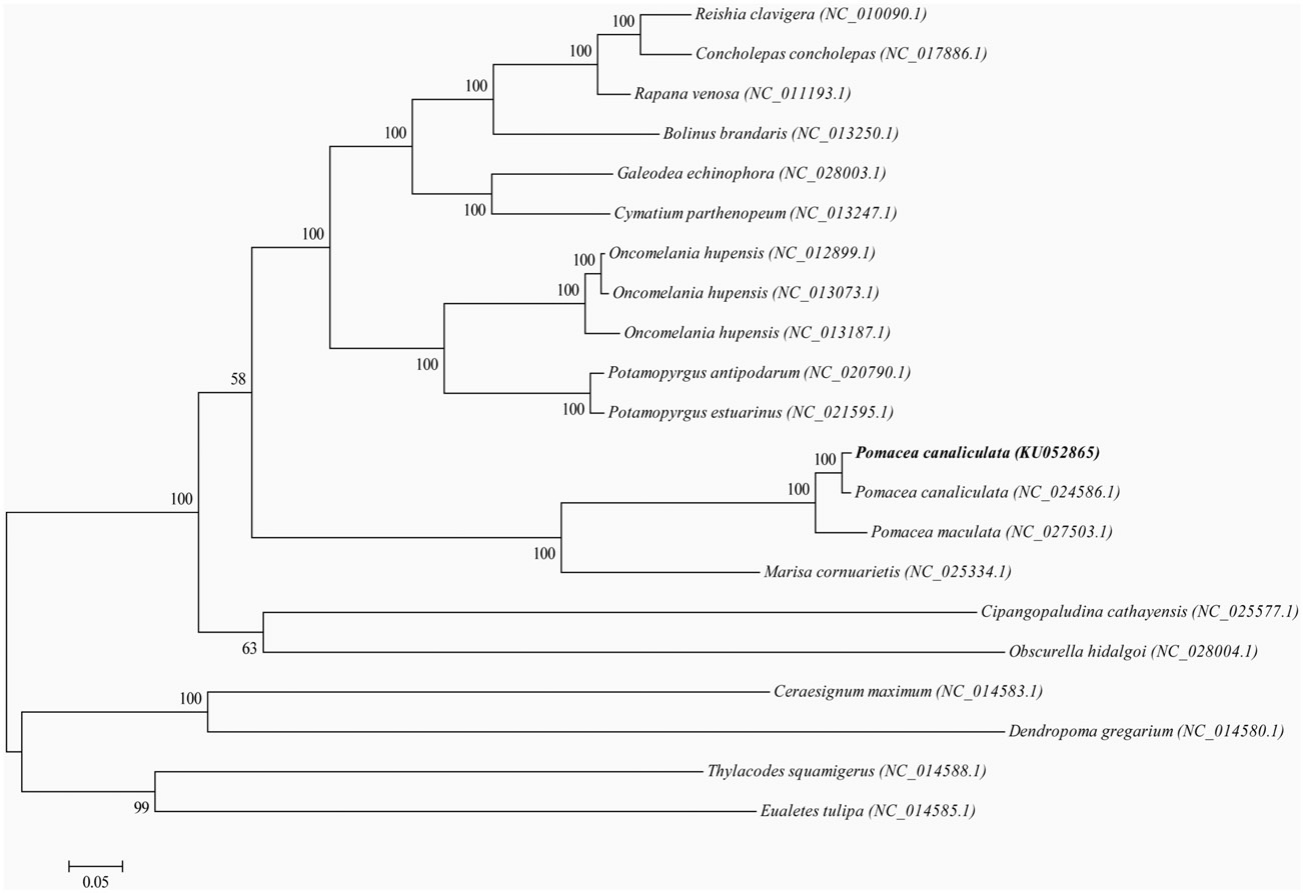
Phylogenetic tree generated by the maximum-likelihood method based on the complete mitochondrial genomes. The published sequences in GenBank adopted are *Reishia clavigera* (NC_010090.1), *Concholepas concholepas* (NC_017886.1), *Rapana venosa* (NC_011193.1), *Bolinus brandaris* (NC_013250.1), *Galeodea echinophora* (NC_028003.1), *Cymatium parthenopeum* (NC_013247.1), *Oncomelania hupensis* (NC_012899.1), *Oncomelania hupensis* (NC_013073.1), *Oncomelania hupensis* (NC_013187.1), *Potamopyrgus antipodarum* (NC_020790.1), *Potamopyrgus estuarinus* (NC_021595.1), *Pomacea canaliculata* (KU052865), *Pomacea canaliculata* (NC_024586.1), *Pomacea maculata* (NC_027503.1), *Marisa cornuarietis* (NC_025334.1), *Cipangopaludina cathayensis* (NC_025577.1), *Obscurella hidalgoi* (NC_028004.1), *Ceraesignum maximum* (NC_014583.1), *Dendropoma gregarium* (NC_014580.1), *Thylacodes squamigerus* (NC_014588.1), *Eualetes tulipa* (NC_014585.1).
